# An elevated level of interleukin-17A in a Senegalese malaria cohort is associated with rs8193038 IL-17A genetic variant

**DOI:** 10.1186/s12879-024-09149-8

**Published:** 2024-03-04

**Authors:** Fatou Thiam, Gora Diop, Cedric Coulonges, Celine Derbois, Alassane Thiam, Abou Abdallah Malick Diouara, Mame Ndew Mbaye, Mamadou Diop, Cheikh Momar Nguer, Yakhya Dieye, Babacar Mbengue, Jean-Francois Zagury, Jean-Francois Deleuze, Alioune Dieye

**Affiliations:** 1 Groupe de Recherche Biotechnologies Appliquees & Bioprocedes Environnementaux, Ecole Superieure Polytechnique, Universite Cheikh Anta Diop de Dakar, Corniche Ouest, Dakar-Fann, BP: 5085 Senegal; 2 Departement de Biologie Animale, Faculte Des Sciences Et Techniques, Unite Postulante de Biologie GenetiqueGenomique Et Bio-Informatique (G2B), Universite Cheikh Anta DIOP, Avenue Cheikh Anta DIOP, Dakar, BP: 5005 Senegal; 3 Pole d’Immunophysiopathologie & Maladies Infectieuses (IMI), Institut Pasteur de Dakar, 36, Avenue Pasteur, Dakar, BP: 220 Senegal; 4 Equipe GBA «GenomiqueBioinformatique & Applications», Conservatoire National Des Arts Et Metiers, 292, Rue Saint Martin, Paris Cedex 03, Paris 75141 France; 5 Centre National de Recherche en Génétique Humaine (CNRGH), Institut de Biologie François Jacob, 2 Rue Gaston Crémieux, CP 5721, Evry Cedex, 91057 France; 6 Pôle de Microbiologie, Institut Pasteur de Dakar, 36 Avenue Pasteur, Dakar, BP 220 Senegal; 7 Service d’Immunologie, Faculté de Médecine, de Pharmacie Et d’Odontostomatologie, Université Cheikh Anta DIOP, Avenue Cheikh Anta DIOP, Dakar, BP: 5005 Senegal

**Keywords:** *Plasmodium falciparum*, Cytokines, Single nucleotide polymorphism, Severe malaria

## Abstract

Malaria infection is a multifactorial disease partly modulated by host immuno-genetic factors. Recent evidence has demonstrated the importance of Interleukin-17 family proinflammatory cytokines and their genetic variants in host immunity. However, limited knowledge exists about their role in parasitic infections such as malaria. We aimed to investigate IL-17A serum levels in patients with severe and uncomplicated malaria and gene polymorphism’s influence on the IL-17A serum levels. In this research, 125 severe (SM) and uncomplicated (UM) malaria patients and 48 free malaria controls were enrolled. IL-17A serum levels were measured with ELISA. PCR and DNA sequencing were used to assess host genetic polymorphisms *in IL-17A*. We performed a multivariate regression to estimate the impact of human *IL-17A* variants on IL-17A serum levels and malaria outcomes. Elevated serum IL-17A levels accompanied by increased parasitemia were found in SM patients compared to UM and controls (*P* < 0.0001). Also, the IL-17A levels were lower in SM patients who were deceased than in those who survived. In addition, the minor allele frequencies (MAF) of two IL-17A polymorphisms (rs3819024 and rs3748067) were more prevalent in SM patients than UM patients, indicating an essential role in SM. Interestingly, the heterozygous rs8193038 AG genotype was significantly associated with higher levels of IL-17A than the homozygous wild type (AA). According to our results, it can be concluded that the IL-17A gene rs8193038 polymorphism significantly affects IL-17A gene expression. Our results fill a gap in the implication of *IL-17A* gene polymorphisms on the cytokine level in a malaria cohort. IL-17A gene polymorphisms also may influence cytokine production in response to Plasmodium infections and may contribute to the hyperinflammatory responses during severe malaria outcomes.

## Background

Malaria is an infectious disease, potentially fatal, caused by Plasmodium protozoan parasites and transmitted by female Anopheles mosquitoes. According to the WHO, malaria is one of the leading causes of death worldwide. Globally, there were an estimated 247 million malaria cases in 2021 in 84 malaria-endemic countries, increasing from 245 million in 2020, with most of this increase coming from countries in the African Region. Furthermore, in 2020, malaria deaths increased by 10% compared with 2019, and between 2019 and 2021, there were 63,000 deaths due to disruptions to essential malaria services during the COVID-19 pandemic. Thus, malaria remains a major public health problem, especially in Africa [1]. In Senegal, Malaria is endemic throughout, and the entire population is exposed to the disease. In 2020, 0.7% of malaria deaths worldwide occurred in Senegal. The number of malaria cases fell by 4.4% between 2017 and 2020, from 52 to 50 per 1,000 inhabitants at risk, while the number of malaria deaths rose slightly by 1.8% over the same period, from 0.24 to 0.245 per 1,000 inhabitants at risk.

The malaria pathogenesis is complex and needs to be elucidated. During blood-stage infection, the host’s immune system produces proinflammatory cytokines to eliminate the parasite, including IL-6, IFN-γ, and TNF, which are pivotal in controlling the parasite’s growth and elimination. In many studies, the high levels of some pro-inflammatory cytokines have been protective in malaria [2–4]. Pro-inflammatory biomarkers were more elevated in cerebral malaria than in non-cerebral malaria patients [5]. Regulatory cytokines such as transforming growth factor-β (TGF-β) and IL-10 balance the pro-inflammatory and anti-inflammatory responses. However, in many cases, cytokines have a double role. On the one hand, they contribute to parasitic clearance; on the other, they are responsible for pathological changes encountered in malaria. Cytokine-modulating strategies may represent a promising modern approach to disease management [3], and the Circulating levels of cytokines have the potential to be biomarkers for severity or protection against malaria.

Recently, the IL-17 proinflammatory cytokine has gained attention among malaria researchers because of its protective role in immunity against extracellular pathogens [6–8] and for the clearance of intracellular pathogens [9–11]. In addition to its essential role in protective immunity, IL-17 is critical in the pathogenesis of various autoimmune inflammatory diseases. IL-17 is a cytokine family that plays a vital role in innate and adaptive immune systems [12–15]. The IL-17 gene is located on chromosome 6p12, comprises three exons and two introns and is coded with six protein members (IL-17A-F). IL-17A is the most essential member of the IL-17 family. The IL-17 receptor family now comprises 5 members (IL-17RA, RB, RC, RD and RE) [16–18].

In mice, it has been demonstrated that elevated IL-17 levels and high IL-4, IL-12α and IFN-γ levels may be a marker of protection against *Plasmodium berghei* [19]. However, the role of IL-17 in human malarial infection outcomes is poorly described, even if increased IL-17 levels in vivax and falciparum malaria and disease severity have been reported [5, 20]. Further studies are needed to evaluate the implication of IL-17 cytokines' circulation levels and association in malaria protection and/or pathogenesis.

Single Nucleotide Polymorphisms (SNPs) in encoding regions of the *IL-17A* gene may influence changes in its expression and, potentially, malaria pathogenesis and risk. *IL-17A* SNPs have been linked to several malignancies, including gastric and breast cancer [21, 22]. However little is known about the association between *IL-17* gene variation and malaria.

To understand the specific IL-17A roles in malaria infection, we conducted this work to analyse IL-17A levels in a retrospective Senegalese cohort, including healthy controls and severe and uncomplicated malaria subjects. Then, the *IL-17A* gene and its flanking regions were sequenced in individual samples. The SNPs were analysed among individuals concerning malaria disease status to detect their influence on the IL-17A serum levels and its potential associations with malaria severity.

## Methods

### Study participants

Our study was conducted in Senegal, a malaria-endemic country in the Sahelian zone of West Africa. Malaria patients were enrolled using a retrospective cohort from the Hospital Principal of Dakar between the 2012–2015 period and corresponded to subjects with Plasmodium-positive Quantitative Buffy Coat (QBC) [23, 24]. These patients were classified into Uncomplicated Malaria (UM) and Severe Malaria (SM) groups, according to the criteria defined by Saissy et al. 2003 and previously described in [25]. The inclusion criteria for these patients were: firstly, black Senegalese individuals with *P. falciparum* infection confirmed in diagnosis; secondly, persons born in Senegal whose parents and grandparents were born in Senegal; and finally, individuals who have not travelled in the last three months of their hospital admission. The healthy control subjects corresponded to the exposed and uninfected subjects group in the same areas and inclusion criteria. They correspond to patients visiting the hospital for annual health check-up with a Plasmodium-negative QBC test.

The study protocol was approved by UCAD’s Committee on Research and Ethics (CER), which considers that the research proposed respects the appropriate ethical standard and, as a result, approves its execution under “Protocole 0344/2018/CER-UCAD”. Written informed consent was obtained from adult participants and parents or legal representatives of children.

The study included 48 CTR, 54 UM and 71 SM subjects. The study’s objectives have been explained clearly using the local dialect before including patients in hospital centres.

### Serum collection and IL-17A quantification

Blood samples were collected from malaria patients and controls and drawn into EDTA vacutainer tubes. Samples were centrifuged, plasma aliquoted, and stored at − 20 °C until testing. Sandwich quantified serum levels of IL-17A enzyme-linked immunosorbent assays (ELISA) using the pre-designed kit (Elabscience Biotechnology ®, USA) as per the manufacturer. The sensitivity of the assay protocol was 2.38 pg/mL, and the coefficient of variation (CV) for intra-assays and inter-assays was < 8% and < 10%, respectively. The micro ELISA plates provided in the kit have been pre-coated with an antibody specific to Human IL-17A. Standards and samples were added to the micro ELISA plate wells and combined with the specific antibody. Then a biotinylated detection antibody specific for Human IL-17A and Avidin-Horseradish Peroxidase (HRP) conjugate were added successively to each microplate well and incubated. The wells were washed 3 times with 350 uL of wash buffer, and the substrate solution was added to each well. Only those wells that contain Human IL-17A, biotinylated detection antibody and Avidin-HRP conjugate will have appeared blue in colour. The enzyme–substrate reaction was terminated by adding a stop solution, and the colour turned yellow. The optical density (OD) was measured spectrophotometrically at a wavelength of 450 nm ± 2 nm. The OD value was proportional to the concentration of Human IL-17A. We have calculated the concentration of Human IL-17A in the samples by comparing the OD of the samples to the standard curve. The sensitivity of the assay protocol was 18.75 pg/mL, and the coefficient of variation (CV) for intra-assays and inter-assays was < 10%.

### Genotyping

DNA was extracted from the peripheral blood of each subject by using standard Qiagen Kits according to the manufacturer’s recommendations. The concentration and quality of the extracted DNA were measured using a NanoDrop spectrophotometer (Thermo Fisher Scientific, Waltham, MA, USA). The *IL-17A* polymorphisms were genotyped using the polymerase chain reaction method. The Oligonucleotide primers used to amplify promoters and exon regions are listed in Table 1. The PCR reactions were performed using a Gotaq®Green Master Mix (Promega, Germany) in a total volume of 25 µl containing 25 ng of genomic DNA (5 ng/µl) and 2.5 µL of each primer (10 μM). The PCR conditions were initial denaturation at 95 °C for 5 min, 35 cycles at 95 °C for 30 s, 60 °C for 30 s, and 72 °C for 5 min, with a final extension at 72 °C for 10 min. The amplicons were purified using BioGel P100 gels (Bio-Rad). Sequencing reactions (2 µL of PCR product) were performed using the dye terminator v3.1 method in an ABI PRISMs 3730 DNA Analyzer (Applied Biosystems, Foster City, CA, USA). Sequencing conditions were: 96 °C for 5 min, 25 cycles of 96 °C for 10 s, 60 °C for 4 min and 15 °C forever, and PCR products were purified with Sephadex G50 superfine columns (GE Healthcare). Alignment of acquired sequences and SNP discovery were performed using *NC_000006.12* as a reference. Analysis was performed with Genalys version 2.0b software [26].

**Table 1:** List of Primers used to amplify the exons of IL17A by polymerase chain reaction (PCR)

PRIMERS	Sequence (5′ > 3′)	Primer Orientation	Product Lenght (bp)
IL17APromPF	CCAAGTTGCTTGGTAGCATG	Sense	557
IL17APromPR	CAGTGGGTTCAGGGGTGACA	Antisense	
IL17AEx1PF	TAGCAGCTCTGCTCAGCTTC	Sense	563
IL17AEx1PR	CTTCTTGTGTGGTTTAGCCC	Antisense	
IL17AEx3_1PF	TTGGTCTTCTTCTGTCTGTC	Sense	291
IL17AEx3_1PR	AGTCAAACCTTCCTTCTTGG	Antisense	
IL17AEx3_3PF	ATAATGGCCCTGAGGAATGG	Sense	554
IL17AEx3_3PR	ACCCCTGGATTTGGAATAGG	Antisense	

*PF* Primer Forward, *PR* Primer Reverse, *bp* base pair

### Statistical analysis

GraphPad Prism v9.5.1 was employed for all statistical analyses. One-way variance analysis ANOVA or Student statistical tests compared serum levels of IL-17A in malaria groups and controls. We calculated Allelic frequencies and Hardy–Weinberg equilibrium, as described previously (Rodriguez, Gaunt & Day, 2009) [27]. Then, as reported previously, the differences in allelic frequencies between the three groups (SM, UM, CTR) were determined using the logistic regression analysis method [28, 29]. Next, associations between *IL-17A* genotypes and serum levels and malaria outcomes were performed using the Mann–Whitney test, and then associations with *P* values < 0.05 were considered statistically significant.

## Results

### Characteristics of malaria patients and healthy controls

A total of 125 malaria patients and 48 controls were included in this retrospective study. The clinical characteristics of the malaria and control groups are summarized in Table 2. The mean values of all haematological parameters included in our study were determined in three categories of enrolled subjects, and the statistical analysis was done using an ANOVA test. A significant difference was observed in parameters while comparing malaria cases and controls, such as age, haemoglobin level, blood cell parameters and leukocyte cells. However, there were no statistically significant differences in monocytes, Basophils. We then compared the same parameters between severe and uncomplicated malaria patients using the Student's t-test. We found the same results as above except for lymphocytes and neutrophils, for which the differences in means between SM and UM were insignificant.

**Table 2:** Baseline characteristics of malaria patients and healthy controls. The means of selected parameters were compared in three groups using the ANOVA test (*P* value^a^) or in two groups, SM and UM using the Student test (*P* value^b^). Values were statistically significant when *p* < 0.05

Characteristics	CTR (*n* = 48)	UM (*n* = 54)	SM (*n* = 71)	*P* value^a^	*P* value^b^
Age, (M ± SD, year)	30.54 (16.14)	13.72 (17.56)	20.76 (20.17)	*P* < 0.0001	0.07
Gender, (%)					
Male	21 (43.75)	19 (35.20)	37 (52.11)		
Female	27 (56.25)	31 (57.40)	31 (43.67)		
Hb (M ± SD, g/dl)	12.95 (1.930)	12.49 (2.855)	8.631 (3.150)	*P* < 0.0001	*P* < 0.0001
Hematocrit (M ± SD, %)	38.76 (5.762)	37.65 (8.382)	25.22 (9.631)	*P* < 0.0001	*P* < 0.0001
MCV (M ± SD, fL)	83.25 (5.687)	88.60 (12.90)	79.14 (13.25)	*P* = 0.0009	*P* = 0.007
RBCs (M ± SD, ^×^ 10^6^/µL)	4.699 (0.632)	4.145 (0.796)	3.149 (1.162)	*P* < 0.0001	*P* < 0.0001
MCHC (M ± SD, pg/cell)	33.60 (0.626)	33.74 (3.159)	33.33 (1.536)	*P* = 0.6685	*P* = 0.289
Leucocyte (M ± SD, ^×^ 10^3^/µL)	5.933 (2.405)	8.217 (4.351)	12.40 (6.952)	*P* < 0.0001	*P* = 0.0008
Neutrophil (M ± SD, %)	43.71 (10.64)	59.95 (15.36)	54.05 (25.60)	*P* = 0.0068	*P* = 0.766
Lymphocyte (M ± SD, %)	43.21 (10.89)	29.27 (13.13)	22.86 (17.03)	*P* < 0.0001	*P* = 0.538
Monocyte (M ± SD, %)	7.970 (2.887)	7.312 (5.597)	6.662 (4.691)	*P* = 0.5191	*P* = 0.663
Eosinophil (M ± SD, %)	4.210 (3.829)	3.2 (2,815)	0.461 (0.451)	*P* < 0.0001	*P* = 0.0002
Basophil (M ± SD, %)	0.8925 (0.558)	0.717 (0.575)	0.6858 (0.729)	*P* = 0.3369	*P* = 0.239

*M* ± *SD* mean ± Standard Deviation, *CTR* Control, *UM* Uncomplicated Malaria, *SM* Severe Malaria, *Hb* Hemoglobin, *MCV* Mean Corpuscular Volume, *RBCs* Red Blood Cells, *MCHC* Mean Corpuscular Hemoglobin Concentration

### Severe malaria patients displayed higher serum IL‑17A compared to uncomplicated malaria and controls

**Fig. 1:**
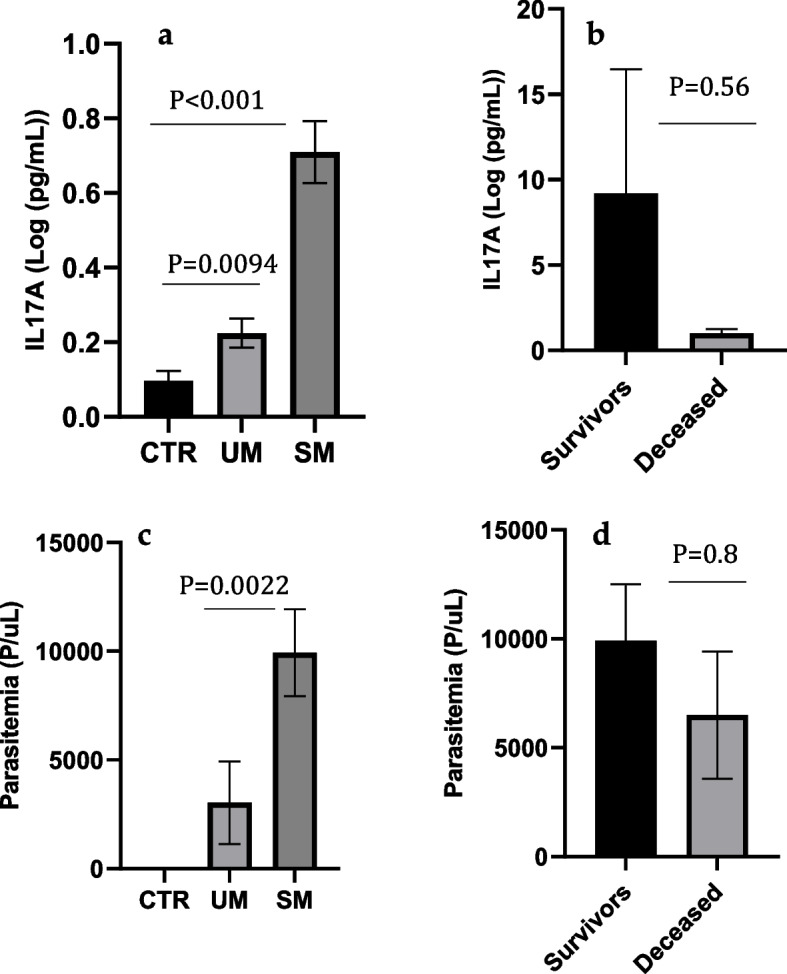
Parasite density and IL17A levels in different categories of enrolled subjects. **a**, **b** Serum level IL-17 between SM, UM and Control group and survivors and deceased SM patients. **c**, **d** Parasitemia between SM, UM and Control group, survivors, and deceased SM patients. Data representing mean pg/ml ± SE for IL17A and mean P/uL for parasitemia were analysed with one way ANOVA or Student Test for comparison. *P* < 0.05 was considered statistically significant

ELISA test quantified the level of IL-17A. As shown in Fig. 1, the mean level of IL-17A was significantly higher in the SM group (mean ± SE: 37.74 ± 140 pg/mL) compared to that of the UM and control groups (mean ± SD:1.69 ± 2.4 pg/ml and 0.67 ± 1.07 pg/mL, respectively; *P* < 0.001) (Fig. 1a). In the SM group, we observed a lower level of IL-17A in patients who were deceased compared to those who survived (mean ± SD: 25.92 ± 46.48 and 40.78 ± 150.67 pg/mL, respectively). Still, the difference was not significant (*P* = 0.56); the lack of significance may be partly due to the small sample size (Fig. 1b). The parasitemia at the time of diagnosis in SM patients was higher than that of the UM patients (mean ± SD: 9039.54 ± 17891 and 3084 ± 14092, respectively; *P* = 0.0022). In addition, there was a decrease in parasitemia in the deceased compared to the survivors, but the difference was not significant (mean ± SD: 9915.54 ± 19185 and 6496 ± 7161, respectively; *P* = 0.8) (Fig. 1c, d).

### Distribution of IL-17A polymorphisms and association with the risk of severe malaria outcome

**Fig. 2:**
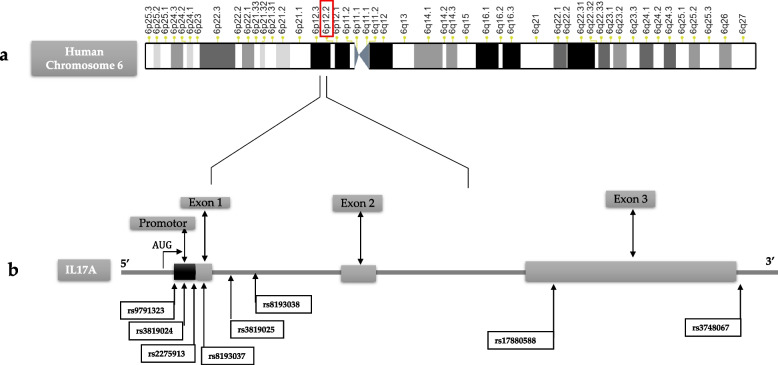
Mapping of Interleukin -17A (IL-17A) gene and the polymorphisms genotyped. Schema of the gene encoding Interleukin 17 A (IL17A) (a) The human IL17A gene (located at 6p12-2) contains 3 exons that span 4 kb. Exons are described by number. AUG: the transcription start site. Black arrows mark eight specific polymorphisms identified in the IL-17A gene

We analyzed genetic variations on the *IL-17A* gene in the promoter region and all the coding regions by sequencing and identified eight SNPs, including 4 SNPs located in the 5’UTR: *IL-17A* + 521A/C (rs9791323), *IL-17A* + 606A/G (rs3819024), *IL-17A* + 849G/A (rs2275913), *IL-17A* + 973G/A (rs8193037); two SNP located within intron 1: *IL-17A* + 1090G/A (rs3819025), *IL-17A* + 1198A/G (rs8193038); one located within exon 3: *IL-17A* + 3840G/A (rs17880588) and one located in the 3′UTR: *IL-17A* + 5151C/T (rs3748067) (Fig. 2).

The Minor Allele frequencies (MAF) of the eight SNPs loci of the *IL-17A* gene were identified using the Hardy–Weinberg equilibrium test (Table 3). Among them, 6 SNP (rs9791323, rs3819024, rs2275913, rs3819025, rs17880588 and rs3748067) were detected with high frequencies (with MAF > 3%), unlike 2 other SNPs (rs8193037 and rs8193038) with MAF < 3% were observed (Table 3). First, comparisons were performed among the three groups to test whether polymorphisms were associated with malaria severity. Then, statistical *IL-17A* polymorphisms analysis was performed using logistic regression tests with an adjustment for potential confounders such as Hb polymorphisms. The SNPS rs3819024 and rs3748067 yielded a significant association with Severe Malaria. For SM vs UM, the *P* = 0.007 (OR 2.61, 95% CI 0.35– 0.91) and *P* = 0.04 (OR 0.32, 95% CI 1–2.1), respectively. In addition, the SNP rs9791323 was associated with uncomplicated malaria *P* = 0.045 (OR 3.78, 95% CI 2.13 – 7.8) with higher MAF in UM (11.3%) patients compared to controls (3.3%).

**Table 3:** Minor Allele Frequencies of single nucleotide polymorphism (SNP) of *IL17-A* gene and association analysis with severe outcomes. The *p* values for statistical tests were performed using each polymorphism's linear regression model analysis. Association analysis of IL-17A was served separately, and corrections to each other were applied to reflect the real effect of each. Analysis has been carried out by comparing SM vs UM, UM vs CTR, and SM vs CTR. Borderline (0.05 ≤ *p* ≤ 0.1) and significant (0 −  ≤ *p* ≤ 0.05) *p* values are in bold. *The OR (odds ratio) and CI (Confidence intervals) were shown when *p* values were significant for malaria (SM)

**SNP**	**Gene location**	**NCBI dbSNP number**	**MAF**				**HWE, exact *P*-value**		
			CTR	UM	SM	Global	SM vs UM	UM vs CTR	SM vs CTR
**+ 521 (A > C)**	5 Prime UTR	**rs9791323**	0.033	0.113	0.075	0.075	0.37	**0.045** 3.78 (2.13 – 7.8)*	0.2477
**+ 606 (A > G)**	5 Prime UTR	**rs3819024**	0.239	0.113	0.25	0.203	**0.007** 2.61 (1.16 – 5.21)*	0.02	0.876
**+ 849 (G > A)**	5 Prime UTR	rs2275913	0.053	0.028	0.079	0.055	0.15	0.48	0.59
**+ 973 (G > A)**	5 Prime UTR	rs8193037	0.021	0.009	0.024	0.018	0.63	0.60	1
**+ 1090 (G > A)**	intron_variant	rs3819025	0.085	0.104	0.095	0.096	0.83	0.81	1
**+ 1198 (A > G)**	intron_variant	rs8193038	0.021	0.009	0.024	0.018	0.63	0.60	1
**+ 3840 (G > A)**	coding_sequence	rs17880588	0.053	0.034	0.043	0.043	1	0.67	1
**+ 5151 (C > T)**	3 Prime UTR Variant	**rs3748067**	0.125	0.055	0.155	0.112	**0.04** 0.32 (0.15 – 0.90)*	0.10	0.54

*SM* Severe Malaria, *UM* Uncomplicated Malaria, *CTR* Control group, *MAF* Minor Allele Frequency, *HWE* Hardy–Weinberg

### Association of SNP IL-17A + 1198 A/G (rs8193038)) and serum IL-17A concentration

**Fig. 3:**
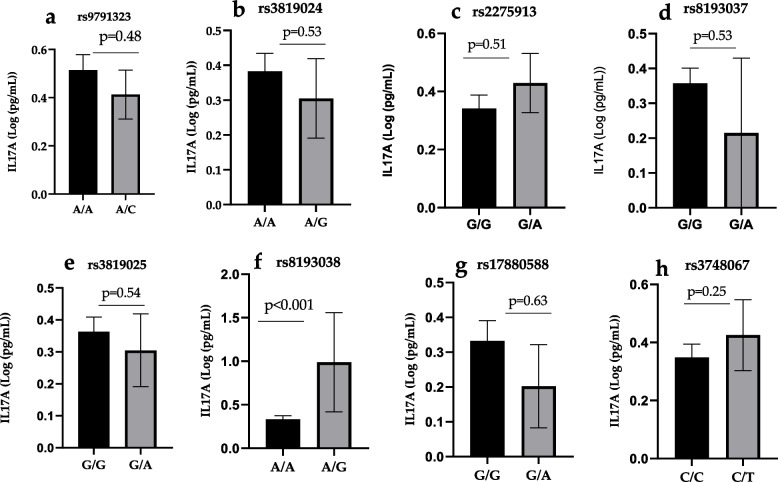
Concentration of IL-17A according to its genotypes. ELISA quantified serum levels of IL-17A in a total of 173 subjects comprising Severe and Uncomplicated malaria subjects and Controls. Mean IL-17A levels in the different genotypes of rs2275913 (**a**) rs3819024 (**b**) rs2275913 (**c**) rs81933037 (**d**) rs3819025 (**e**) rs8193038 (**f**) rs17880588 (**g**) rs3748067 (**h**) Polymorphisms were compared by ANOVA followed by Tukey’s post-test. No significant association between IL-23A (rs11171806) and levels of IL-23 was observed (**b**). A *p*-value of less than 0.05 was taken as significant

The relationship between the *IL-17A* polymorphisms and serum IL-17A concentration was analysed regardless of the differences in the study groups. We compared IL17A levels between the different genotypes of the polymorphisms analysed in the study and found differences in levels depending on genotype. We found that some genotypes were associated with increased IL17A levels (Fig. 3c, f, h), while the opposite was true for others (Fig. 3a, b, d, e, g).

However, only the heterozygous rs8193038 AG genotype is significantly associated with higher levels of IL-17A amongst the whole study groups compared to the homozygous rs8193038 AA genotype (OR = 4.9, 95% CI = (2.01- 8.13), *P*< 0.001).

## Discussion

Analysis of the genetic effects of inflammatory response gene variants such as inflammatory cytokines is a key step in malaria research to understand the underlying mechanisms of pathogenesis. This knowledge will be essential for identifying specific therapies to prevent mortality or adverse complications associated with severe malaria and the long-term consequences that represent a heavy burden in endemic regions [30]. It is understood that cytokine gene polymorphisms could affect the serum levels of cytokines by influencing transcriptional regulation. The IL-17 cytokine family is a relatively new family linked to adaptive and innate immune systems. IL-17A are members of the IL-17 cytokine family, essential for the pathogenic activity of IL-17 cells and the production of various proinflammatory mediators in the body [9, 31]. IL-17A is a multifunctional cytokine which has a protective role in immunity for the clearance of intracellular pathogens such as *Plasmodium spp.* [9–11, 19, 32, 33], making it a good therapeutic biomarker in malaria diseases. However, the role of IL-17A in malaria has not yet been extensively investigated. In addition, the association of common polymorphisms with malaria predisposition and cytokine levels was never analysed in Senegal.

Thus, the present study explored IL-17A cytokine levels and gene polymorphism’s influence on IL-17A serum levels. In recent years, evaluating SNPs has been considered a common approach for testing the impact of human genetic variation on diseases [34].

We have determined the serum IL-17A levels and genotyped *IL-17A* variants in Senegalese severe and uncomplicated malaria patients and controls. We observed elevated IL-17A levels in SM patients compared to the UM and healthy cases. Moreover, the high parasitemia in the SM group accompanied the IL-17A increase. This indicates that IL-17A has an essential regulatory role in malaria infection, controlling the intensity of the immune response, as described in the experimental model, as well as human malaria and several other infectious diseases [5, 19, 35, 36]. IL-17 production is associated with a very high occurrence of chronic inflammation and immunopathological conditions [31]. Recent data suggest that IL-17 contributes to host protection against diverse infectious organisms during sepsis while inducing hyperinflammation with detrimental outcomes for the host under certain conditions [37]. Earlier investigations in the experimental model have deciphered the essential role of IL-17. In *P. vivax* infection, authors suggest that increasing serum IL-17 levels in malaria patients could be considered a host adaptation mechanism to control changes in blood viscosity, and IL-17 could thus be used as an immunomodulatory agent [38]. IL-17 appears to act on erythrocytes by remodelling their cell membrane; it is well-known that erythrocytes in malaria are very sensitive to osmotic shock [38].

We found an elevated level of IL-17A in severe malaria patients who were survivors compared to those who were deceased. Our results seem to confirm the results of Helegbe et al., which showed elevated IL-17 levels together with high IL-4, IL-12α, and IFN-γ levels may be a marker of protection, and the mechanism may be controlled by host factors [19]. Thus, pro-inflammatory IL-17A cytokine seems to have been protective against fatal malaria. Furthermore, the data agree with the observations of Oyegue-Liabagui et al. [20], who noted a correlation between Th17 cell count and overall survival in patients with malaria in children.

Immuno-genetic variants are associated with diverse degrees of malaria susceptibility, including cytokine gene polymorphisms that modify their expression and circulating protein levels to reflect inflammatory or anti-inflammatory responses [39–41]. Polymorphisms in the IL-17A cytokine can impact the activity and expression of inflammatory mediators, which can affect interleukin-17 activity [42, 43]. *IL-17A* gene polymorphisms have been linked to several malignancies, including gastric and breast cancer [21, 22]. However little is known about the association between *IL-17* gene variation and malaria. In this study, we performed a genetic analysis of the variations of the *IL-17A* gene. We identified 8 SNPs in the *IL-17A* gene; among them, 6 SNPs (rs9791323, rs3819024, rs2275913, rs3819025, rs17880588 and rs3748067) were detected with high frequencies (with MAF > 3%) at opposite to 2 other SNPs (rs8193037 and rs8193038) with MAF < 3% were observed. Then, statistical *IL-17A* polymorphism analysis was performed using logistic regression to test whether polymorphisms were associated with malaria severity. For the first time, we identified 2 SNPs associated with severe malaria and one associated with uncomplicated malaria. We found that the SNP rs3748067 reduced the risk of severe malaria (odds ratio (OR) = 0.32; *P* = 0.04). Instead, the SNP rs3819024 was associated with an increased risk of severe malaria (odds ratio (OR) = 2.61; *P* = 0.007). Then, the SNP rs9791323 was associated with a high risk of uncomplicated malaria (odds ratio (OR) = 3.78; *P* = 0.045). We *IL-17A* rs3819024 G could also be considered a biomarker of malaria severity.

Our data reinforce our knowledge of the genetic variants of the IL-17 cytokine family and their potential roles in malaria. Even though the involvement of *IL-17A* variants has yet to be fully elucidated, a previous study had shown that *IL-17F* (rs6913472 and rs4715291) and *IL-17RA* (rs12159217 and rs41396547) polymorphisms independently modulate susceptibility to Cerebral Malaria and provide evidence that IL-17F protects against it [44]. The role of SNPs in malaria disease and immunological disorders has been previously reported [45–47]. Associations between cytokine polymorphisms and malaria support that cytokine gene polymorphisms have an unquestionable role in the orchestration of the immune response, leading to different functional scenarios, which in turn influence the outcome of malaria disease establishment and evolution [20, 48, 49].

The relationship between the *IL-17A* polymorphisms and serum IL-17A concentration was analysed. The SNPs rs3748067, rs3819024 and rs9791323 did not show an association with malaria outcome and IL-17A level. Interestingly, we found that the heterozygous rs8193038 AG genotype is significantly associated with higher levels of IL-17A amongst the whole study groups compared to the homozygous rs8193038 AA genotype (OR = 4.9, 95% CI = (2.01- 8.13), *P* < 0.001). This data suggests that rs8193038 polymorphism significantly affects *IL-17A* gene expression. However, this SNP is located at the intron 1 region, which could correspond to a splicing site and explain our result [50, 51]. It is well known that the introns regulate gene expression; they contain enhancers or other cis-acting elements that promote the initiation or elongation of transcription. Introns are also involved in alternative splicing and genome imprinting. Intron splicing increases mRNA stability in the nucleus [52]. It has been shown that intronal SNPs regulate protein synthesis by mRNA splicing [53]. In addition, functional SNPs in introns are sometimes linked to SNPs in neighbouring genes, influencing mRNA splicing, among other things [54]. The genome-wide analysis of human SNPs near splice sites revealed 1300 SNPs, which are probably capable of modifying the protein by changing splicing [55]. Further studies are needed to elucidate how this intronic rs8193038 SNP influences mRNA splicing and IL-17A expression.

Our results fill a gap in the implication of *IL-17A* gene polymorphisms on the cytokine level in a Senegalese malaria cohort. *IL-17A* gene polymorphisms also may influence cytokine production in response to *Plasmodium* infections and may be contribute to the hyperinflammatory responses during severe malaria outcomes. A series of studies performed in the last decade emphasized the *IL-17A* SNPs, particularly the rs2275913 variant, and serum cytokines levels in numerous pathogenesis. A recent study by Lang et al. revealed the association between the *IL-17A* rs2275913 variant with higher cytokine serum levels and predisposed Preeclampsia development in Chinese patients [56]. In leprosy, Farag et al*.* have demonstrated that IL17A rs2275913 genotype GG was associated with significantly increased IL-17A levels in Egyptian patients [57]. Finally, another study by Li et al. revealed that *IL-17A* polymorphisms may influence hepatocellular carcinoma risk in chronic hepatitis B virus infection via regulating IL-17A production [58]. However, our study found no association between rs2275913 variant and serum IL-17A levels, even if a high serum level of IL-17A was associated with the heterozygous rs2275913 GA genotype. The lack of significance may be due to a limited number of subjects. These relationships could likely reach statistical significance in a larger cohort of patients. Our results suggest that the association between *IL-17* gene polymorphisms and serum levels may depend on ethnic group populations and/or pathogenesis mechanisms. Then, we found that different IL-17A alleles play different roles in immunity, producing different cytokine levels and disease outcomes, as reported in other studies [59].

## Conclusions

The current report revealed an elevated level of IL-17A in severe malaria patients compared to healthy controls in Senegalese cohort. Furthermore, heterozygous mutant and minor alleles of *IL-17A* rs3819024 and rs3748067 polymorphisms predisposed subjects for the development of SM. Interestingly, the current report further validated the functional relevance of *IL-17A* (rs8193038) variants and demonstrated the association of mutants with elevated IL-17A levels. However, further studies, including more significant sample sizes in the different populations, are required to validate the observations of the present study. In addition, further investigation on the role of IL-17 and its interplay with other immune factors needs to be conducted in clinical settings. In the future, we plan to carry out a mechanistic study to understand the role of these *IL-17A* SNPs in gene expression. We intend to measure the expression of IL-17A mRNA to confirm our results. We will use a genome-wide association study to screen the IL-17A gene better and characterize the position of the SNPs better. Finally, we will perform proteomic studies and use bioinformatics tools to understand the molecular function of SNPs.

## Data Availability

The data and materials supporting the conclusions of the study are available from the corresponding author on request.

## Abbreviations

COVID: Coronavirus disease 2019
CTR: Control
EDTA: Ethylene Diamine Tetraacetic Acid
ELISA: Enzyme-Linked Immuno Assay
IFN: Interferon
IL: Interleukin
MAF: Minor Allele Frequency
PCR: Polymerase Chain Reaction
SM: Severe Malaria
SNP: Single Nucleotide Polymorphism
TGF: Tumor Growth Factor
TNF: Tumor Necrosis Factor
UCAD: Universite Cheikh Anta Diop
UM: Uncomplicated Malaria
WHO: World Health Organization
